# Variability in gastrointestinal multiplex PCR panel ordering in urgent care: a potential diagnostic stewardship target

**DOI:** 10.1128/spectrum.02382-25

**Published:** 2025-10-14

**Authors:** Timothy C. Jenkins, Melody A. Zwakenberg, Margaret Cooper, Katherine C. Shihadeh, Michael Breyer, Cory K. Hussain, Laura Triplett, Lindsey E. Fish

**Affiliations:** 1 Department of Medicine, Division of Infectious Diseases, Denver Health 47804, Denver, Colorado, USA; 2 Department of Emergency Medicine, Denver Health 47804, Denver, Colorado, USA; 3 Department of Pharmacy, Denver Health 47804, Denver, Colorado, USA; 4 Department of Medicine, Sutter Health, San Francisco, California, USA; 5 Department of Pathology and Laboratory Medicine, Denver Health 47804, Denver, Colorado, USA; 6 Department of General Internal Medicine, Denver Health 47804, Denver, Colorado, USA; Icahn School of Medicine at Mount Sinai, New York, New York, USA

**Keywords:** diagnostic stewardship, diagnostic excellence, diarrhea, urgent care

## LETTER

The BioFire FilmArray gastrointestinal multiplex PCR panel (GI panel) is an increasingly used syndromic rapid diagnostic test (1). Because of its high sensitivity, use of this test when there is a high pre-test probability for acute gastroenteritis or when antimicrobial therapy would be indicated if certain targets are detected is essential to ensure results are likely to reflect disease rather than colonization and to prevent unnecessary antimicrobial use. At our institution, however, we noted the GI panel was frequently ordered in the absence of true diarrhea and in low-yield clinical scenarios such as mild or brief symptoms. Understanding utilization patterns and identifying drivers of variability in ordering practices may inform diagnostic stewardship interventions to promote the most appropriate use of the test. The objectives of this study were to evaluate variability in GI panel ordering among urgent care clinicians and assess factors associated with outlier utilization.

We conducted a retrospective study of adult patients with an encounter at two urgent care centers in an integrated healthcare system from 1 July 2022 to 30 June 2024. Distinct groups of physicians, advanced practice providers (APPs), and medical residents staffed the two sites; no provider worked at both sites. During the study period, GI panel ordering was unrestricted, with no standardized criteria for use. The primary outcome was the GI panel order rate, defined as the number of GI panel orders divided by the number of total patient encounters, expressed as a percentage. The order rate was calculated overall, by site (A vs B), provider type (APP vs physician), clinician experience (<5 years vs ≥5 years since training), and by individual provider. Orders by residents were attributed to the supervising physician. Order rates were compared between groups using the χ^2^ test. Outlier high utilization was defined as an individual order rate of two or more standard deviations above the mean.

**Fig 1:**
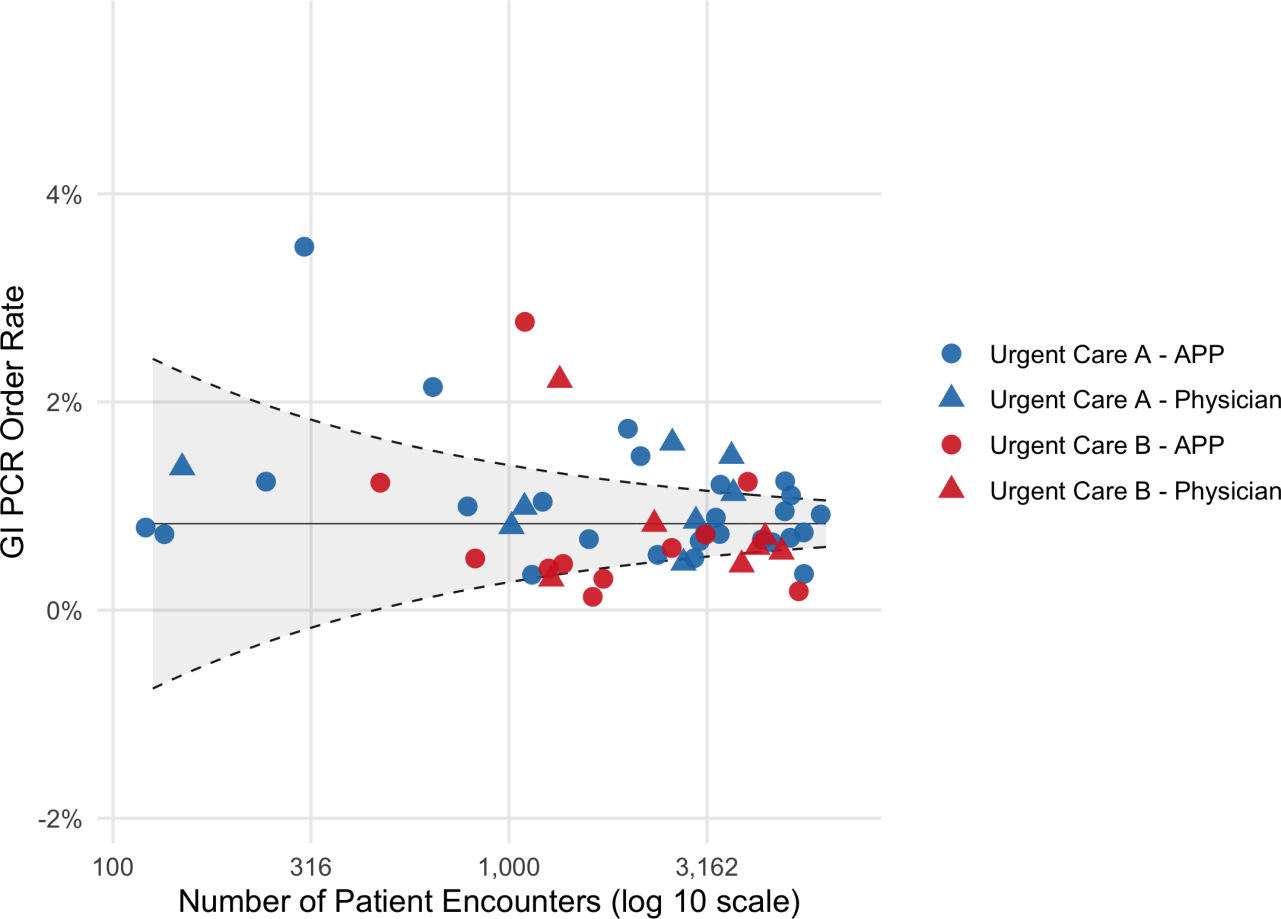
Individual clinician GI PCR panel order rate by volume of encounters. The solid line represents the mean order rate for all providers, and the dashed lines represent the upper and lower control limits at two standard deviations from the mean.

Among 53 clinicians, provider types and years of experience were similar across the two sites (Table S1). Of 142,805 total patient encounters during the study period, 1,186 GI panels were ordered (overall rate of 0.8%). The order rate was significantly higher at Urgent Care A vs B (0.9 vs 0.7%, respectively, *P* < 0.001). APPs and physicians ordered at similar rates (0.8% vs 0.9%, *P* = 0.36). Clinicians with <5 years of experience had a significantly higher order rate than those with ≥5 years of experience (1.0% vs 0.8%, *P* < 0.001). A funnel plot revealed substantial variability in individual order rates both by site and by provider type (Fig. 1). The range of order rates was 0.3–3.5% at Urgent Care A and 0.1–2.8% at Urgent Care B. In total, 10 (19%) clinicians were outlier high utilizers, accounting for 25% of all orders. The proportion of clinicians who were outlier utilizers was similar at both sites (Table S1).

This study has several limitations. First, the data are from a single institution, which limits generalizability. Second, the clinical appropriateness of GI panel orders was not evaluated; however, numerous studies have demonstrated high variation in diagnostic test ordering is indicative of inappropriate use rather than clinical differences (2–4). Despite the limitations, this work highlights the opportunity to couple syndromic multiplex PCR panels with standardized criteria for use to promote their application in appropriate clinical scenarios, which may reduce inter-provider variability in ordering. Our data also suggest that targeting outlier utilizers or high-utilization groups (e.g., newer clinicians) may be an effective diagnostic stewardship approach to drive down low-yield or unnecessary testing.

## SUPPLEMENTAL MATERIAL

Table S1 spectrum.02382-25-s0001.docx Provider characteristics by urgent care site.

ASM does not own the copyrights to Supplemental Material that may be linked to, or accessed through, an article. The authors have granted ASM a non-exclusive, world-wide license to publish the Supplemental Material files. Please contact the corresponding author directly for reuse.
