# Supplementary material for: Variability in gastrointestinal multiplex PCR panel ordering in urgent care: a potential diagnostic stewardship target
Source: Microbiol Spectr. 2025 Oct 14;13(11):e02382-25. doi: 10.1128/spectrum.02382-25 (PMC12584696; doi:10.1128/spectrum.02382-25)
Supplement: Table S1 — Provider characteristics by urgent care site. [file spectrum.02382-25-s0001.docx]

**Supplemental Materials**

**Variability in Gastrointestinal Multiplex PCR Panel Ordering in Urgent Care: A Potential Diagnostic Stewardship Target**

**Supplemental Table.** Clinician characteristics by urgent care site

|  | Urgent Care A  N = 35  n (%) | Urgent Care B  N = 18  n (%) |
| --- | --- | --- |
| Provider type |  |  |
| Advanced practice provider | 27 (77) | 11 (61) |
| Physician | 8 (23) | 7 (39) |
| Years of experience |  |  |
| Less than 5 years | 11 (31) | 6 (33) |
| 5 or more years | 24 (69) | 12 (67) |
| Outlier high utilizer ^a^ | 7 (20) | 3 (17) |

^a^ Defined as an individual order rate of two or more standard deviations above the mean
